# Selection of a compatible electron transport layer and hole transport layer for the mixed perovskite FA_0.85_Cs_0.15_Pb (I_0.85_Br_0.15_)_3_, towards achieving novel structure and high-efficiency perovskite solar cells: a detailed numerical study by SCAPS-1D†

**DOI:** 10.1039/d3ra02170j

**Published:** 2023-06-07

**Authors:** Md. Bulu Rahman, Noor-E-Ashrafi, Md. Helal Miah, Mayeen Uddin Khandaker, Mohammad Aminul Islam

**Affiliations:** a Department of Physics, Bangabandhu Sheikh Mujibur Rahman Science and Technology University Gopalganj 8100 Bangladesh; b Centre for Applied Physics and Radiation Technologies, School of Engineering and Technology, Sunway University 47500 Bandar Sunway Selangor Malaysia; c Department of Electrical Engineering, Faculty of Engineering, Universiti Malaya, Jalan Universiti 50603 Kuala Lumpur Malaysia

## Abstract

The first and foremost intent of our present study is to design a perovskite solar cell favorable for realistic applications with excellent efficiency by utilizing SCAPS-1D. To ensure this motive, the detection of a compatible electron transport layer (ETL) and hole transport layer (HTL) for the suggested mixed perovskite layer entitled FA_0.85_Cs_0.15_Pb (I_0.85_Br_0.15_)_3_ (MPL) was carried out, employing diver ETLs such as SnO_2,_ PCBM, TiO_2_, ZnO, CdS, WO_3_ and WS_2_, and HTLs such as Spiro-OMeTAD, P3HT, CuO, Cu_2_O, CuI, and MoO_3._ The attained simulated results, especially for FTO/SnO_2_/FA_0.85_Cs_0.15_Pb (I_0.85_Br_0.15_)_3_/Spiro-OMeTAD/Au, have been authenticated by the theoretical and experimental data, which endorse our simulation process. From the detailed numerical analysis, WS_2_ and MoO_3_ were chosen as ETL and HTL, respectively, for designing the proposed novel structure of FA_0.85_Cs_0.15_Pb (I_0.85_Br_0.15_)_3_-based perovskite solar cells. With the inspection of several parameters such as variation of the thickness of FA_0.85_Cs_0.15_Pb (I_0.85_Br_0.15_)_3_, WS_2,_ and MoO_3_ including different defect densities, the novel proposed structure has been optimized, and a noteworthy efficiency of 23.39% was achieved with the photovoltaic parameters of *V*_OC_ = 1.07 V, *J*_SC_ = 21.83 mA cm^−2^, and FF = 73.41%. The dark *J*–*V* analysis unraveled the reasons for the excellent photovoltaic parameters of our optimized structure. Furthermore, the scrutinizing of QE, *C*–*V*, Mott–Schottky plot, and the impact of the hysteresis of the optimized structure was executed for further investigation. Our overall investigation disclosed the fact that the proposed novel structure (FTO/WS_2_/FA_0.85_Cs_0.15_Pb (I_0.85_Br_0.15_)_3_/MoO_3_/Au) can be attested as a supreme structure for perovskite solar cells with greater efficiency as well as admissible for practical purposes.

## Abstract 2

Nomination of compatible ETL and HTL for the absorber layer FA_0.85_Cs_0.15_Pb (I_0.85_Br_0.15_)_3_*via* numerical analysis.

## Graphical abstract

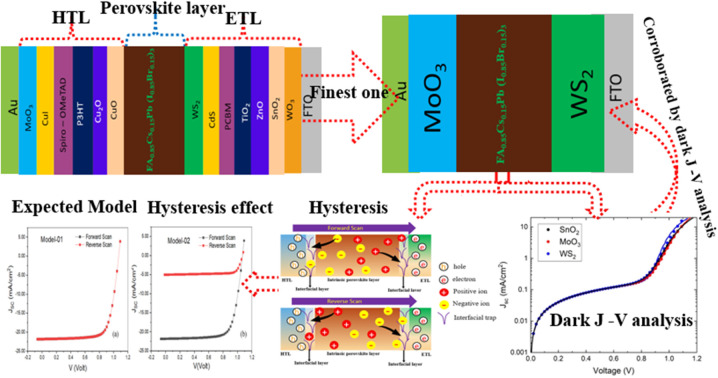

## 1. Introduction

Perovskites with formula AMX_3_ (where A, M, and X are organic/inorganic cations, metal cations, and oxygen/halogen anions, respectively) are extremely lucrative materials in the sector of photovoltaic technology owing to their intriguing optoelectronic properties such as tunable band gap, high absorption coefficients, large charge carrier diffusion lengths and high mobility, and ambipolar charge transportation.^1,2^ Kojima *et al.*^3^ first utilized a perovskite material named CH_3_NH_3_PbI_3_ for photovoltaic applications and obtained a power conversion efficiency (PCE) of 3.81%. Perovskite material-based solar cells are gaining substantial attention in terms of efficiency, and throwing challenges to the existing solar cells including silicon-based solar cells. Recently, Min *et al.*^4^ reported 25.8% PCE by employing FAPbI_3_-based solar cells. However, Si-based solar cells are still overshadowing other PSCs due to their high PCE (26.7%^5^) as well as longevity. In addition to this, a group of scientists from Helmholtz-Zentrum Berlin fabricated tandem solar cells by adopting silicon and perovskite material and attained a world record PCE of 32.5%, which ultimately glorifies the necessity of perovskite material. The composition of the stoichiometric structure of perovskite AMX_3_ governs the energy band gap, crystal phase, stability as well as performance in photovoltaic applications. The Goldschmidt geometric tolerance factor, 
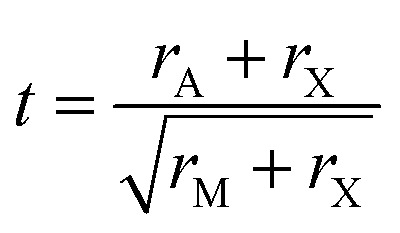
 where *r*_A_, *r*_M_ and *r*_X_ are the effective ionic radii for A, M and X, respectively, determines the formability and stability of a perovskite structure.^6^ The tolerance factor can be aligned within 0.8–1.0 for steady and worthy structure in photovoltaic technology by partially or fully supplanting the ions of various sites in the AMX_3_ structure. In a recent study, Liu *et al.*^7^ synthesized a perovskite solar cell (PSC) of structure FTO/NiO_*x*_/CsPbI_3_/ZnO/ITO in which the MA cation was substituted by Cs in the perovskite material. They reported a PCE of 16.04%, which was capable to retain 90% PCE even after 3000 hours of constant lighting and heating. In another study, Saliba *et al.*^8^ fabricated mixed perovskite structures of Cs_*x*_(MA_0.17_FA_0.83_)_1−*x*_Pb(I_0.83_Br_0.17_)_3_ and obtained a PCE of 21.1% by utilizing this structure in a PSC. The reported structure was able to hold nearly 97% of its initial efficiency after 250 hours of light exposure.

In another extensive study carried out by Liu *et al.*^9^ where they developed a PSC by applying Cs_0.05_FA_0.79_MA_0.16_PbI_2.49_Br_0.51_ perovskite structure and claimed that 93% of the initial PCE (21.1%) was maintained under humidity (30–60%) for 1200 hours.

Besides the perovskite material, an efficient PSC has a dependency on ETL, HTL, and the interfacial ETL/perovskite layer and perovskite layer/HTL layers.^10^ Semiconducting materials are reckoned as charge transport materials that render better transparency in the solar spectrum, broad bandgap, high mobility of charge carriers, and greater thermal and chemical stability. ETL, which extract photo-generated electrons from the perovskite layer and precludes the transfer of hole towards the electrode *via* ETL, and eventually electron–hole recombination at the interfaces of the perovskite layer gets inhibited. One of the crucial features of selecting ETL is that the energy levels of the material should be compatible with perovskite material to extract electrons and preclude holes. Normally, n-type semiconducting materials such as ZnO, SnO_2_, CdS, TiO_2_, WO_3,_ WS_2_, PCBM, and TMD are broadly esteemed as the ETL. By replacing or modifying the ETL, the overall stability and performance of the PSC can be greatly influenced. Pathak *et al.*^11^ modified the ETL of the device Spiro-OMeTAD/CH_3_NH_3_PbI_3_/TiO_2_ by doping it with Al (0.3 mol%), and subsequently, PCE was increased by approximately 24%. Another analysis was conducted by Song *et al.*,^12^ in which, TiO_2_ was replaced with SnO_2_ of the architecture Spiro-OMeTAD/CH_3_NH_3_PbI_3_/TiO_2_ and substantiated 13% PCE. They also emphasized that PSC with SnO_2_-based solar cells are more stable in comparison with TiO_2_ based solar cells. In a recent study, Min *et al.*^4^ achieved highest PCE (25.8%) by employing SnO_2_ as an ETL.

HTL provides a pivotal role in extracting photo-generated holes from the perovskite layer and acts as a blocking element for electrons toward the metal electrode. One of the important aspects of choosing HTL is that the energy levels of the material should be relevant to perovskite material for the purpose of hole extraction and blocking the electron. Usually, HTL is a p-type semiconducting material including Spiro-OMeTAD, MoO_3_, Cu_2_O, PEDOT:PSS, CuSCN, CuI, PTAA, CuO, and P3HT.

**Fig. 1:**
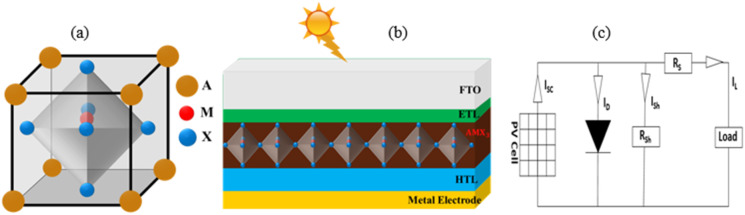
A schematic diagram of (a) perovskite structure, (b) n–i–p structure of PSC, and (c) equivalent circuit of the PSC.

The first ever solid-state HTL (Spiro-OMeTAD) was utilized by Kim^20^*et al.* in Kojima's first perovskite, and enhancement of PCE from 3.8% to 9.7% was observed. By substituting or modifying the HTL layer, the overall performance of the PSC can be influenced to some extent. By applying MoO_3_ instead of PEDOT:PSS and WO_3_ as the HTL, Tseng *et al.* synthesize PSCs with the active layer of CH_3_NH_3_PbI_3_ and obtained 13.1% PCE.^21^ Their analysis distinctively disclosed that in terms of overall performance, the devices manufactured with the MoO_3_ film are better than the devices employed with WO_3_ and PEDOT:PSS as the HTL. Since MoO_3_ has high work function (6.9 eV), better conductivity (1.2 × 10^−7^ sm^−1^) as well as a larger band-gap (>3 eV, no absorption in the visible-infrared range), it has established itself as a competent HTL.^22^ A schematic diagram of the perovskite structure, n–i–p structured PSC, and equivalent circuit of the PSC are portrayed in Fig. 1.

In this theoretical work, we have executed a computational analysis by utilizing a solar cell capacitance simulator (SCAPS 1D-3.3.1.0) for choosing compatible ETL and HTL with FA_0.85_Cs_0.15_Pb (I_0.85_Br_0.15_)_3_ (MPL) as the mixed perovskite layer. For this motive, primarily, we selected SnO_2,_ PCBM, TiO_2_, ZnO, CdS, WO_3,_ and WS_2_ as ETLs. Likewise, Spiro-OMeTAD, P3HT, CuO, Cu_2_O, CuI, and MoO_3_ were chosen as HTLs in our proposed n–i–p structure. All initial input physical parameters (IIPPs) of ETL, HTL, and MPL utilized in this simulated study were collected from the previously reported experimental and simulated data. Our prime focus for this research was to identify the structure that serves the best performance. Herein, 42 structures have been formulated by using the aforementioned ETLs and HTLs with MPL, and eventually, the preferred one was found as well. Finally, we have probed the effect of changing thickness, the defect density of MPL, and interfacial defects on our best structure (FTO/WS_2_/MPL/MoO_3_/Au). In addition, the impact of ETL's shallow uniform donor density on the *C*–*V* and M–S plots of the optimized structure was scrutinized. Moreover, QE analysis and the hysteresis effect of the optimized structure were dissected. Finally, the dark *J*–*V* analysis was executed to unravel the reasons behind the tremendous photovoltaic parameters of our optimized structure. To the best of our knowledge, this is the first-ever comprehensive study on this unique structure.

## 2. Computational details

SCAPS, developed by the University of Gent, is a one-dimensional and proficient solar cell simulator among other simulators (SILVACO, ATLAS, AMPS, COMSOL, *etc*)^23–25^ and it is widely used to realize the essence of energy band structure, *I*–*V* characteristics, *C*–*V* analysis, M−S analysis, doping profile, C-f study, spectral response, *etc.*, of a solar cell. SCAPS-1D is governed by three fundamental eqn (i)–(iii) named continuity equations for holes and electrons, and Poisson's equations to fortuitously complete its task under equilibrium conditions.i
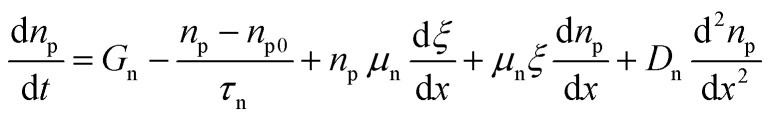
ii
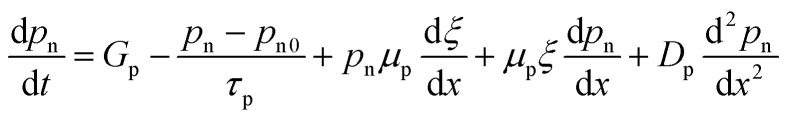
iii
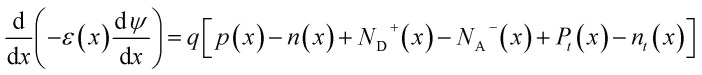
where *ξ* represents the electric field, *ε* represents permittivity, *q* is the electronic charge, *τ* is the carrier lifetime, charge mobilities are indicated by *μ*, shallow acceptor and shallow donor concentrations are represented *N*_A_ and *N*_D_, respectively, *D* represents the diffusion coefficient of the charge carrier, the charge carrier generation rate is indicated by *G*, and defect densities of electrons and holes are mentioned by *n*_*t*_(*x*) and *p*_*t*_(*x*).

## 3. Results and discussions

In a previous study, Karthick *et al.*^13^ synthesized FTO/SnO_2_/FA_0.85_Cs_0.15_Pb(I_0.85_Br_0.15_)_3_/Spiro-OMeTAD/Au-based PSC structure and obtained *V*_OC_ = 1 V, *J*_sc_ = 22.6 mA cm^−2^, FF = 64.4% and PCE = 15.1%. We simulated this structure by using SCAPS to corroborate and compare it with the experimental results. In this simulation, we considered the realistic facts by inserting bulk defects, amphoteric defects, interfacial defects, resistance, *etc.*, and the rest of the IIPPs of the different layers were rendered from different published studies, as shown in Table 1. We maintained the following data in all defect layers for all simulations: the defect type was neutral, energetic distribution was Gaussian, characteristic energy was 0.1 eV, and energy level with respect to reference 0.6 eV and electron/hole capture cross-section 10^−15^ cm^2^. We also added interfacial defects at the MPL/ETL and MPL/HTL layers with 2 × 10^11^ cm^−3^ and 2 × 10^10^ cm^−3^, respectively, in which the electron/hole capture cross section was 10^−19^ cm^2^. In addition to that, amphoteric defect of 2 × 10^15^ cm^−3^ was included in the proposed MPL. Furthermore, we performed the simulation by considering constant light illumination of 1000 Watt per m^2^ at 1.5 AM ranging from 300–1000 at a temperature of 300 K, series resistance of 3 Ω cm^2^, and shunt resistance of 5000 Ω cm^2^.

**Table 1:** IIPPs for each layer of the PSC collected from ref. 13

Parameters	FTO	SnO_2_	FA_0.85_Cs_0.15_Pb (I_0.85_Br_0.15_)_3_	Spiro-OMeTAD	Au
Thickness (*t*) in nm	500	70	350	165	—
Band gap (*E*_g_) in eV	3.5	3.5	1.59	2.9	—
Electron affinity (*χ*) in eV	4	4	4.09	2.2	—
Dielectric permittivity (*ε*_r_)	9	9	6.6	3	—
CB effective density of states (*N*_c_) in cm^−3^	2.2 × 10^18^	2.2 × 10^17^	2 × 10^19^	2.2 × 10^18^	—
VB effective density of states (*N*_v_) in cm^−3^	2.2 × 10^18^	2.2 × 10^17^	2 × 10^18^	2.2 × 10^18^	—
Electron thermal velocity (*V*_e_) in cm s^−1^	1 × 10^7^	1 × 10^7^	1 × 10^7^	1 × 10^7^	—
Hole thermal velocity (*V*_h_) in cm s^−1^	1 × 10^7^	1 × 10^7^	1 × 10^7^	1 × 10^7^	—
Electron mobility (*μ*_e)_ in cm^2^ V^−1^ s^−1^	20	20	8.16	0.0001	—
Hole mobility (*μ*_h_) in cm^2^ V^−1^ s^−1^	10	10	2	0.0001	—
Shallow uniform acceptor density (*N*_A_) in cm^−3^	0	0	1.3 × 10^16^	1.3 × 10^18^	—
Shallow uniform donor density (*N*_D_) in cm^−3^	1 × 10^15^	1 × 10^15^	1.3 × 10^16^	0	—
Defect density (*N*_t_) in cm^−3^	1 × 10^18^	1 × 10^18^	4 × 10^13^	1 × 10^15^	—
Amphoteric defect density (ADD) in cm^−3^	—	—	2 × 10^15^	—	—
Work function (*φ*) in (eV)	—	—	—	—	5.3

The obtained photovoltaic parameters (PPs) of the structured PSC (S1) were as follows *V*_OC_ = 0.96 V, *J*_SC_ = 20.03 mA cm^−2^, FF = 53.84% and PCE = 14.12%. This simulated result is consistent with the aforementioned experimental results, which vindicated the validity of our simulation.

**Fig. 2:**
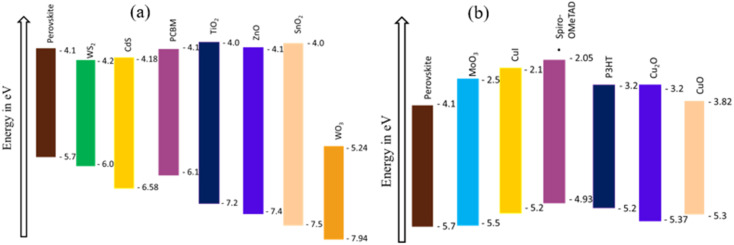
Relative alignment of the energy band of FA_0.85_Cs_0.15_Pb (I_0.85_Br_0.15_)_3_ (MPL) with (a) ETLs and (b) HTLs.^13–19^

To observe the finest structures with performance, 42 structures were constructed and simulated by varying different ETLs and HTLs with the aforementioned proposed MPL. The IIPPs of ETLs and HTLs are tabulated in Tables 1 and 2. The simulated results of the 42 structures are displayed in Tables 3 and TSI 01.† Different values of PPs were demonstrated by dissimilar structures depending on the band alignment (as shown in Fig. 2) of ETLs and HTLs with MPL. The CBO between MPL and ETLs, and VBO between MPL and HTLs indicate the condition of band alignment. For positive/negative values of VBO and CBO, a spike/cliff is established.^35^ In the case of negative VBO, there is no hindrance of flowing holes to the back electrode. However, the increment of negative VBO gives rise to interfacial recombination. On the other hand, for negative CBO, the electron can simply drift to ETL.^36^ However, for positive CBO (0–0.3 eV), low interfacial recombination and selective charge collection occurred.^14^ The CBO and VBO calculated by using the following eqn (iv) and (v) are displayed in Table 4.

iv CBO = *χ*_MPL_ − *χ*_ETL_

v VBO = (*χ*_HTL_ + *E*_gHTL_) − (*χ*_MPL_ + *E*_gMPL_)

where, *χ*_MPL_/*χ*_ETL_/*χ*_HTL_ is the electron affinity of MPL/ETL/HTL, respectively, and *E*_gMPL_/*E*_gHTL_ is band gap of MPL/HTL, respectively. Although CBO between MPL and WO_3_ possesses the highest value than that for CBO between MPL and WS_2,_ better performance was exhibited by the structure with WS_2_ as ETL. With the large band gap as well as the existence of valence band maxima in relatively lower energy as compared to WS_2_, it may create inhibition in hole transportation. As a result, it offers lower performance compared to the structure with WS_2_.

**Table 2:** IIPPs of ETLs and HTLs

ETLs							HTLS				
Parameters	PCBM	TiO_2_	ZnO	CdS	WO_3_	WS_2_	P3HT	CuO	Cu_2_O	CuI	MoO_3_
*t* (nm)	70	70	70	70	70	70	165	165	165	165	165
*E*_g_ (eV)	2	3.2	3.3	2.4	2.6	1.8	2	1.48	2.17	3.1	3
*χ* (eV)	4.2	4.1	4.1	4.18	3.8	3.95	3.2	4.07	3.2	2.1	2.5
*ε*_r_	3.9	9	9	10	4.8	13.6	3	18.1	7.11	6.5	12.5
*N*_c_ (cm^−3^)	2.5 × 10^21^	2.2 × 10^18^	2.2 × 10^18^	2.2 × 10^18^	2.2 × 10^21^	2.2 × 10^17^	1 × 10^20^	2.1 × 10^19^	2 × 10^17^	2.8 × 10^19^	2.2 × 10^18^
*N*_v_ (cm^−3^)	2.5 × 10^21^	1 × 10^19^	1.9 × 10^19^	1.9 × 10^19^	2.2 × 10^21^	2.2 × 10^16^	1 × 10^20^	5.5 × 10^19^	1.1 × 10^19^	1 × 10^19^	1.8 × 10^19^
*V*_e_ (cm s^−1^)	1 × 10^7^	1 × 10^7^	1 × 10^7^	1 × 10^7^	1 × 10^7^	1 × 10^7^	1 × 10^7^	1 × 10^7^	1 × 10^7^	1 × 10^7^	1 × 10^7^
*V*_h_ (cm s^−1^)	1 × 10^7^	1 × 10^7^	1 × 10^7^	1 × 10^7^	1 × 10^7^	1 × 10^7^	1 × 10^7^	1 × 10^7^	1 × 10^7^	1 × 10^7^	1 × 10^7^
*μ*_e_ (cm^2^ V^−1^ s^−1^)	0.2	20	100	100	30	100	1 × 10^−4^	100	200	100	25
*μ*_h_ (cm^2^ V^−1^ s^−1^)	0.2	10	25	25	30	100	1 × 10^−4^	0.1	80	43.9	100
*N*_A_ (cm^−3^)	0	0	0	0	0	0	1 × 10^16^	1 × 10^16^	1 × 10^18^	1 × 10^18^	1 × 10^18^
*N*_D_ (cm^−3^)	2.93 × 10^17^	1 × 10^18^	1 × 10^18^	1 × 10^18^	6.35 × 10^17^	1 × 10^18^	0	0	0	0	0
*N*_t_ (cm^−3^)	1 × 10^14^	1 × 10^14^	1 × 10^15^	1 × 10^15^	1 × 10^15^	1 × 10^15^	1 × 10^15^	1 × 10^14^	1 × 10^15^	1 × 10^15^	1 × 10^15^
Ref.	15	26 and 27	26	28	29	30	27 and 31	15	32	33	34

**Table 3:** Performance of various PSC structures

Name of the structure	*V*_oc_ (V)	*J*_sc_ (mA cm^−2^)	FF (%)	*η* (%)
FTO/SnO_2_/MPL/Spiro-OMeTAD/Au (S1)	0.96	20.09	53.84	14.12
FTO/SnO_2_/MPL/Cu_2_O/Au (S4)	1.09	20.42	64.60	19.64
FTO/SnO_2_/MPL/MoO_3_/Au (S6)	1.08	20.19	68.84	20.40
FTO/TiO_2_/MPL/Cu_2_O/Au (S16)	1.11	20.47	70.58	21.77
FTO/TiO_2_/MPL/MoO_3_/Au (S18)	1.09	20.22	75.13	22.58
FTO/ZnO/MPL/Cu_2_O/Au (S22)	1.11	20.47	70.58	21.77
FTO/ZnO/MPL/MoO_3_/Au (S24)	1.09	20.22	75.13	22.58
FTO/CdS MPL/Cu_2_O/Au (S28)	1.11	20.51	70.31	21.70
FTO/CdS/MPL/MoO_3_/Au (S30)	1.09	20.30	75.03	22.60
FTO/WO_3_/MPL/Cu_2_O/Au (S34)	1.11	20.48	70.59	21.79
FTO/WO_3_/MPL/MoO_3_/Au (S36)	1.09	20.25	75.12	22.61
FTO/WS_2_/MPL/Cu_2_O/Au (S40)	1.11	20.87	70.41	22.16
FTO/WS_2_/MPL/MoO_3_/Au (S42)	1.09	20.71	74.93	23.10

**Table 4:** CBO and VBO of the MPL with ETLs and HTLs

ETL	PCBM	TiO_2_	SnO_2_	ZnO	CdS	WO_3_	WS_2_	HTL	Spiro-OMeTAD	P3HT	CuO	Cu_2_O	CuI	MoO_3_
CBO	−0.11	−0.01	0.09	−0.01	−0.09	0.29	0.14	VBO	−0.75	−0.48	−0.13	−0.31	−0.48	−0.18

From Tables 3 and TSI 01,† we have distinguished S4, S6, S16, S18, S22, S24, S28, S30, S34, S36, S40, and S42 as aspiring structures from 42 formulated structures according to their performance. S42 structure was the most satisfactory one among all the simulated devices, in which WS_2_ and MoO_3_ were utilized as ETL and HTL, respectively, and the obtained PPs were *V*_OC_ = 1.09 V, *J*_SC_ = 20.71 mA cm^−2^, FF = 74.93%, and PCE = 23.10%. For further investigation of our research work, we extensively focused on optimizing the structure S42 by varying the different parameters of the layers.

### 3.1. Thickness optimization

The effect of changing MPL thickness was carried out from 300 nm to 700 nm while thicknesses of both ETL and HTL were sustained at 50 nm. The photovoltaic parameter *J*_SC_ was increased by 17.81% from the initial value. However, FF and *V*_OC_ were dropped 10.88% and 5.1%, respectively, from their original value owing to the change in MPL thickness. The decline meant that *V*_OC_ is happened due to the decrease in dark saturation current with the increase in thickness of the MPL.^37^ In the case of FF, it showed declining behavior due to the increase in series resistance with the rise in the MPL thickness.^38^ The positive gradient of current density at lower thickness is comparatively higher up to 450 nm after that the rate slowed down gradually. With increasing thickness, the absorption of a photon is more and consequently generates more electron–hole pairs. As a result, *J*_SC_ was eventually increased. After 450 nm thickness, rate of increasing *J*_SC_ becomes slow because of the dominating recombination rate.

**Fig. 3:**
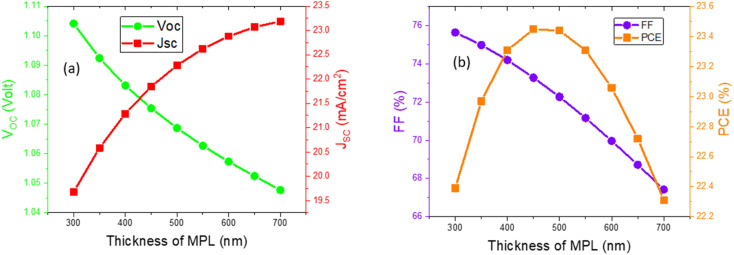
Variation of the device characteristics, such as *V*_OC_, *J*_SC_, FF, and PCE with respect to the thickness of the MPL.

Another important photovoltaic parameter, PCE increases with the increase in thickness of the MPL up to 450 nm at which maximum PCE was observed (23.45%) and then a slumping pattern was noticed till 700 nm. With the increasing thickness after 450 nm, PCE decreases due to the increase in series resistance and the recombination rate may also get higher owing to the lower diffusion length compared to the thickness.^39^ The thickness of the MPL was optimized at 450 nm due to the aforementioned reason and the PPs were 1.08 V, 21.84 mA cm^−2^, 73.28%, and 23.45% for *V*_OC_, *J*_SC_, FF and PCE, respectively. Variations of PPs with respect to MPL thickness are displayed in Fig. 3.

The precise selection of parameters for ETL and HTL is crucial for fabricating highly efficient solar cells. The careful selection of ETL helps to minimize the recombination current and significantly enhances the transmittance, leading to improved overall performance.^40^ To optimize the thickness of ETL, the thickness was changed from 10 nm to 100 nm and the thicknesses of the MPL and HTL were kept constant at 350 nm and 70 nm, respectively. The PCE of the device increased by 2.7% from the initial PCE of 22.66% and the change of other PPs was not a significant amount, which is displayed in Fig. FSI 1.† By considering the feasibility of the fabrication technique, we fixed the optimization value of ETL thickness at 50 nm. At 50 nm, the PPs for *V*_OC_, *J*_SC_, FF and PCE were 1.0924 V, 20.58317 mA cm^−2^, 74.99%, and 22.97% respectively. Similarly, the thickness of the HTL was also varied from 10–100 nm by holding MPL thickness at 350 nm and ETL thickness at 50 nm. Owing to this, the change in PCE was noted slightly up to 50 nm, however, after 50 nm, the value was constant, which is displayed in Fig. FSI 2.† By analyzing the simulated data, the HTL thickness was optimized at 50 nm. From the clear inspection of our results, it is evident that the thickness of ETL and HTL imposes a very slight influence on the performance, which is also justified through previous works.^40,41^

### 3.2. Defects optimization

Defect density can be termed as a dominant parameter that deleteriously influences the performance of the device. The amount of charge carriers that can reach the respective electrode, which is initiated from the perovskite layer, greatly relies on this defect density. The recombination process is expedited with the increasing defect density at the perovskite layer. The relation between the recombination process to the defect density of the perovskite layer is illustrated by the Shockley–Read–Hall (SRH)^42^ recombination model (vi) as follows;vi
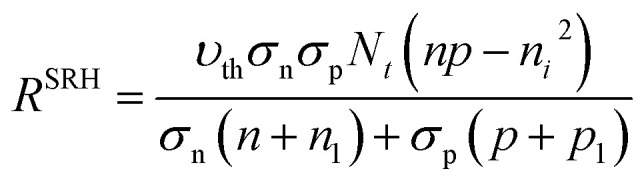
where, *υ*_th_ is the thermal velocity of charge carriers, *σ*_n_/*σ*_p_ is the capture cross-section of electrons/holes, *N*_t_ is the number of defects per unit volume, *n*/*p* is the equilibrium electron/hole concentration, *n*_i_ is the intrinsic carrier concentration and *n*_1_/*p*_1_ is the concentration of electron/holes at trap states/valence band, respectively. The impact of the defect density on the lifetime of the carrier and diffusion length is explicated by the following eqn (vii) and (viii),^43^vii
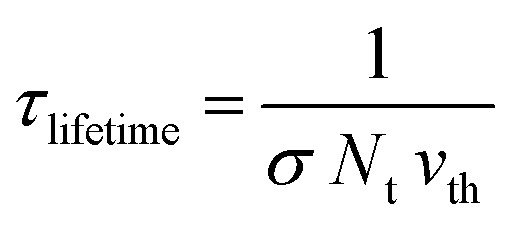
viii
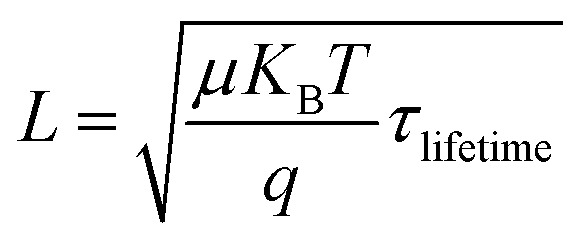
where, *τ*_lifetime_ is the lifetime of the charge carrier, *L* is the charge carrier diffusion length and *μ* is the mobility of the charge carrier.

**Fig. 4:**
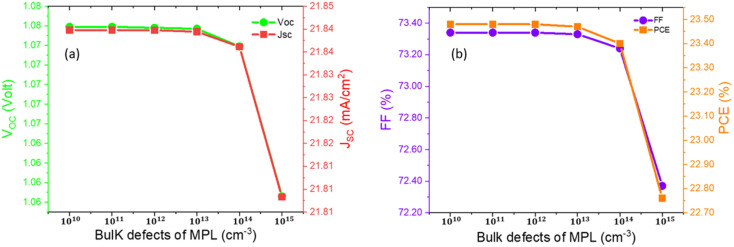
Variation of the device characteristics, such as *V*_OC_, *J*_SC_, FF, and PCE with respect to bulk defects of the MPL.

To examine the impact of bulk defect density of MPL, the defect density was varied from 10^10^ cm^−3^ to 10^15^ cm^−3^. All the PPs showed insignificant change with the bulk defect density and the PPs such as *V*_OC_, *J*_SC_, FF and PCE were changed by 1.6%, 0.17%, 1.32%, and 3.07%, respectively. The PPs were almost constant up to 10^14^ cm^−3^ bulk defect density. Hence 10^14^ cm^−3^ is termed as the tolerable bulk defect density of the structure. After that, the PPs decreased gradually with the increase in the defect density owing to the decrease in diffusion length, which results in increased bulk recombination as explained in the above equation. Variations of PPs owing to changing bulk defect density are shown in Fig. 4. Improving the crystalline quality and having a larger grain size have been proven to be advantageous to lessen the effect of the bulk defect. To perform this, photo-curing and thin-film post-processing can be employed.^44^

Interfacial trap density critically influences the PPs. In a perovskite material having a large absorption coefficient, photo-generated charge carrier numbers are high at the light illumination side compared to the backside. Interface recombination center has a greater impact on the probability of charge collection than severe recombination centers.

**Fig. 5:**
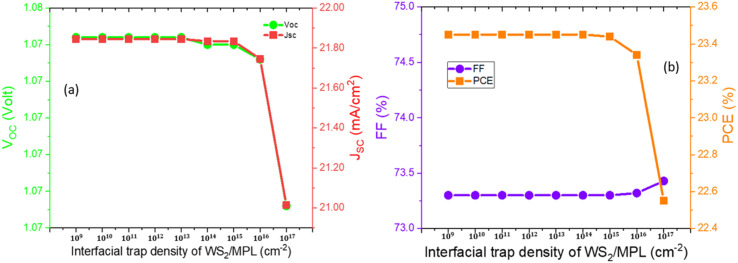
Variation of the device characteristics, such as *V*_OC_, *J*_SC_, FF, and PCE with respect to the interfacial trap density of WS_2_/MPL.

If light enters the perovskite structure through ETL, then the recombination rate at the ETL/perovskite layer interfaces is greater compared to the perovskite layer/HTL interfaces due to the generation of more electron–hole pairs. Poor interface quality severely influences the performance of the PSC. As a consequence of generating more electron–hole pairs in the ETL/perovskite layer interface because of light traveling from ETL to HTL *via* the absorbing layer, the rate of recombination was found higher in the ETL/perovskite layer interface than in the HTL/perovskite layer. For this reason, the ETL/perovskite interfacial layer is more sensitive. To analyze the effect of interfacial defects on the PPs, the interfacial trap density at WS_2_/FA_0.85_Cs_0.15_Pb (I_0.85_Br_0.15_)_3_ was varied from 10^10^ cm^−3^ to 10^17^ cm^−3^. No appreciable change was noticed for the PPs up to 10^15^ cm^−3^. After 10^15^ cm^−3^, PCE starts to decrease gradually. Changes in PPs concerning the interfacial traps of WS_2_/MPL are depicted in Fig. 5.

**Fig. 6:**
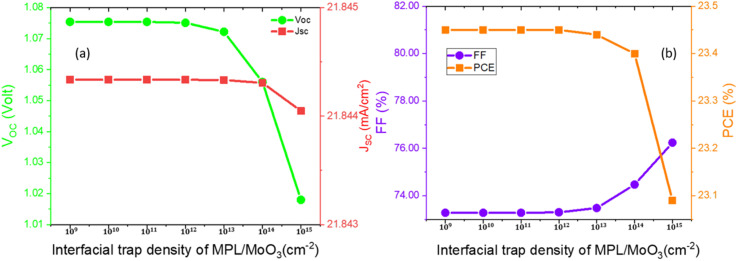
Variation of the device characteristics, such as *V*_OC_, *J*_SC_, FF, and PCE with respect to the interfacial trap density of MPL/MoO_3._

The interfacial trap density at FA_0.85_Cs_0.15_Pb (I_0.85_Br_0.15_)_3_/MoO_3_ was altered as well from 10^10^ cm^−3^ to 10^15^ cm^−3^ to visualize the impact on PPs. Variations of PPs for interfacial defects of MPL/MoO_3_ are depicted in Fig. 6. The PPs remain unchanged up to 10^13^ cm^−3^ and after that, the PPs show a slight reduction. Because of the variation of interfacial defect density at ETL/MPL as well as MPL/HTL, reductions by 3.5% and 1.5% in PCE were detected, respectively. A greater recombination rate is the sole reason for the reduction in PPs with the increase in interfacial defect density. By weighing the above facts, 10^15^ cm^−3^, and 10^13^ cm^−3^ were reckoned as the optimized interfacial trap density at ETL/MPL and MPL/HTL layers, respectively. To improve the band alignment between ETL/MPL and MPL/HTL layers, as well as to eradicate the defects and trap states in the interfacial layer, a buffer layer or additives with ETL or HTL can be a beneficial solution.^45,46^ However, in both cases, the FF is slightly increased at the higher trap densities. To know the essence of this behavior of the FF parameter, further investigation is required.

### 3.3. Impact of defect density and thickness on quantum efficiency

**Fig. 7:**
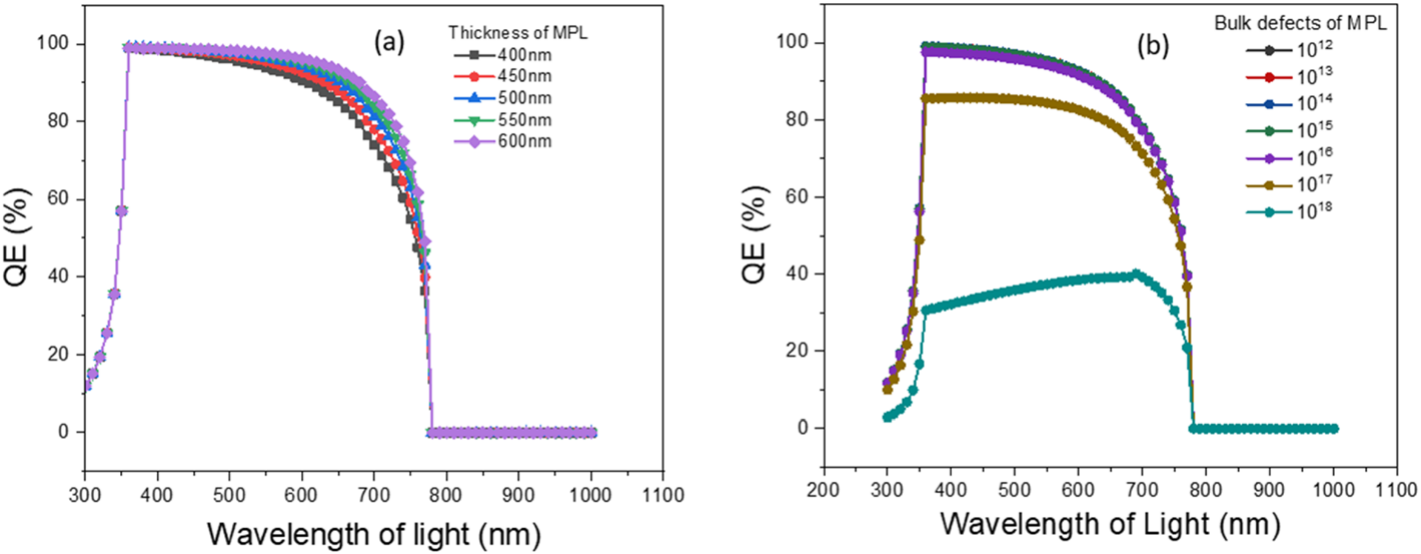
Variation of QE owing to (a) thickness and (b) bulk defects of MPL.

The efficiency of the photovoltaic device can be calculated from IQE and EQE, where IQE refers to the total photocurrent generated under light illumination and EQE implies the optical performance of the PSC.^47–49^ To realize the consequences of differing thickness on quantum efficiency, the MPL thickness was changed and the results are illustrated in Fig. 7a. For a thickness of 300 nm, the maximum quantum efficiency was 97.54% at a wavelength of 360 nm. Thickness range from 400 nm to 600 nm, the maximum quantum efficiency of about 99% was detected at 360 nm. An unnoticeable change of absorption has been observed after the 780 nm wavelength of light. All of our modeled structures showcased a noteworthy amount of QE, for the range of visible light. Our proposed structure having a thickness of 450 nm, 98.8%, and 77.9% QE was assessed at wavelengths of 400 nm and 700 nm, respectively. Greater QE implied a larger collection of charge carriers, which signifies that our simulated structure demonstrated the best performance under visible light. The inspection was executed carefully to realize the outcome of altering the bulk density of MPL on QE by varying the bulk density from 10^12^ cm^−3^ to 10^18^ cm^−3^ and the obtained results are depicted in Fig. 7b. Approximately, 98.5% QE was encountered for defect density of up to 10^16^ cm^−3^ at a wavelength of 360 nm of light. However, the severe slump in QE was found after 10^16^ cm^−3^ and detection of 40% QE at 10^18^ cm^−3^ defect density in which the light wavelength was 690 nm. Unnoticeable absorption was observed after the 780 nm wavelength of light for all defect densities. In our proposed structure, having defect density of 10^14^ cm^−3^, QE values were 99% to 78% at around 400 nm and 700 nm wavelength, respectively. Thus, this structure exhibits the best spectral response under visible light.

### 3.4. Capacitance–voltage characteristics of the PSC

The capacitance–voltage (*C*–*V*) technique can also be regarded as another salient technique for characterizing a solar cell by employing SCAPS-1D. Our present study emphasized exploring the outcome of ETL's *N*_D_ on *C*–*V* as well as the Mott–Schottky plot (M−S). Built-in potential, a foremost parameter, which is necessary for accumulating photo-generated holes and electrons in opposite directions from each other, can be acquired from the M−S plot. The capacitance of a PSC can be correlated with the built-in potential by the following formula (ix),^50^ix
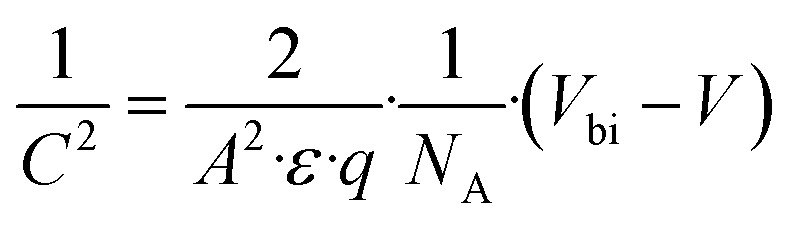
where *V* = applied voltage, *V*_bi_ = built-in potential, *A* = area, *C* = Capacitance, *N*_A_= dopant density, and *ε* = permittivity of the vacuum medium.

Because of the influence of space charge, a larger capacitance value can be attained, which ultimately lowers the value of built-in potential. The recombination rate in a solar cell is notably lowered with the larger built-in potential, therefore, the attainment of greater PCE may be possible. After a definite range of voltage, the slope of capacitance can be negative. This negative capacitance may also be created from recombination or self-heating.

**Fig. 8:**
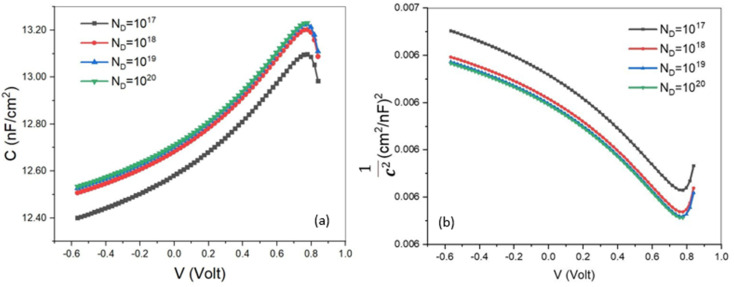
(a) *C*–*V* characteristics and (b) MS plot as a function of ETL's *N*_D_.

To probe the consequences of ETL's *N*_D_ on the *C*–*V* and Mott–Schottky plots, this density was changed from 10^17^ cm^−3^ to 10^20^ cm^−3^ and the obtained results are mapped in Fig. 8. An identical graphical pattern for capacitance against voltage was found for all the varied donor densities, where, capacitance revealed exponentially increasing behavior with voltage until the approximate value of the voltage 0.78 V. This phenomenon can be narrated as a large number of electrons being confined in the space charge region (SCR). In other words, a trivial amount of recombination of electron–hole pairs has happened in that region. After this certain value (0.78 V), capacitance manifested a sudden drop, offering a negative slope. The reason behind this can be ascribed in terms of the transformation of electron traps into hole traps in the SCR. According to the inspection of Burgelman *et al.*^51^ on defect density, the lowering rate of electron density traps is equivalent to the growth rate of hole density traps in the SCR, which implies the alteration of electron traps into hole traps. The maximum capacitances were assessed as 13.23 nF cm^−2^, 13.22 nF cm^−2^, 13.20 nF cm^−2^, and 13.09 nF cm^−2^ for uniform shallow donor densities of 10^20^ cm^−3^, 10^19^ cm^−3^, 10^18^ cm^−3^, and 10^17^ cm^−3^, respectively. From the scrutiny of the Mott–Schottky plot, the built-in potential was attained as 1.0 V to 0.85 V from the approximate intersection point at the horizontal axis for every graph with the *N*_D_ of 10^17^ cm^−3^ to 10^20^ cm^−3^. Photo-generated holes and electrons can efficaciously proceed towards the respective electrode under the dominance of a larger built-in potential that finally provides us with higher conversion efficiency. In accordance with our inspection, the rising of donor density lowers the value of built-in potential owing to the elevation of the trapped electron in SCR that ultimately gives an increment in capacitance.

### 3.5. Hysteresis effect on the performance of the PSC

**Fig. 9:**
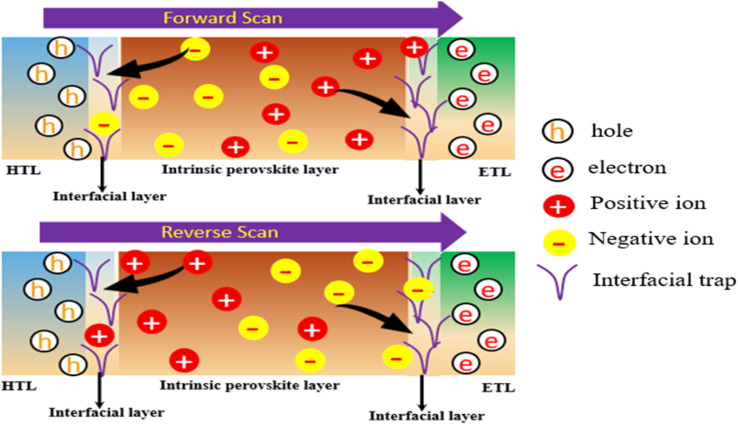
A schematic of the charge migration and re-orientation in the perovskite layer under the influence of forward and reverse scans.

**Fig. 10:**
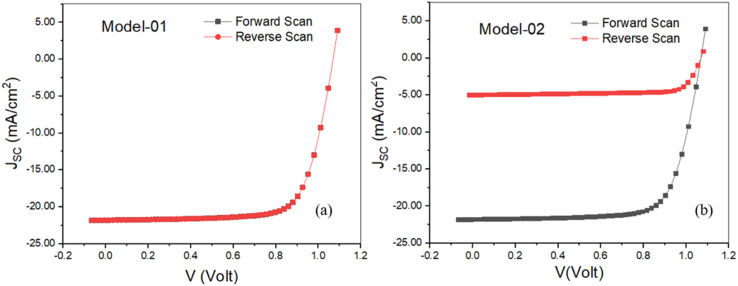
Hysteresis effect on the *J*–*V* characteristics.

Ion migration and ion accumulation at the interfaces (portrayed in Fig. 9) under biasing voltage are one of the aspects involved in creating hysteresis in the *J*–*V* characteristics of a PSC.^52^ Two types of models entitled Model-01 where ETL and HTL defects were termed as donor type and acceptor type defects, respectively, keeping the other defects as neutral type. In Model-02, defects of HTL/MPL, as well as MPL/ETL, were considered acceptor-type defects and donor-type defects, respectively, whereas MPL and FTO defects were considered neutral-type defects. In both cases, forward and reverse scans were examined by switching the defect type to investigate hysteresis behavior in the *J*–*V* curve. No hysteresis has been noticed in model-1, as shown in Fig. 10a. However, in model-2, the severe impact of hysteresis on the *J*–*V* curve was noted, as shown in Fig. 10b. Because of the influence of hysteresis, the amount of *J*_SC_ and PCE dropped to 76.94% and 75.40%, respectively, whereas *V*_OC_ showed a steady value in both cases and a slight increment in FF (6.7%) was pointed out as well.

As the interfacial layer is adjacent to the perovskite layer, ion accumulation and/or migration certainly have a significant influence on carrier transportation from perovskite to ETL and HTL. This could be the reason for generating the hysteresis effect in model 2. It has been reported that the *J*–*V* hysteresis in PSCs is caused by the localization of positively charged ions at the interface between the ETL and the perovskite layer.^53^ Furthermore, the localized ions may accelerate non-radiative recombination, resulting in the rapid deterioration of PSCs.^53–55^ Thus, to assure a reliable structure with the best performance, the proposed model-1 could be the best option.

### 3.6. Dark current analysis

**Fig. 11:**
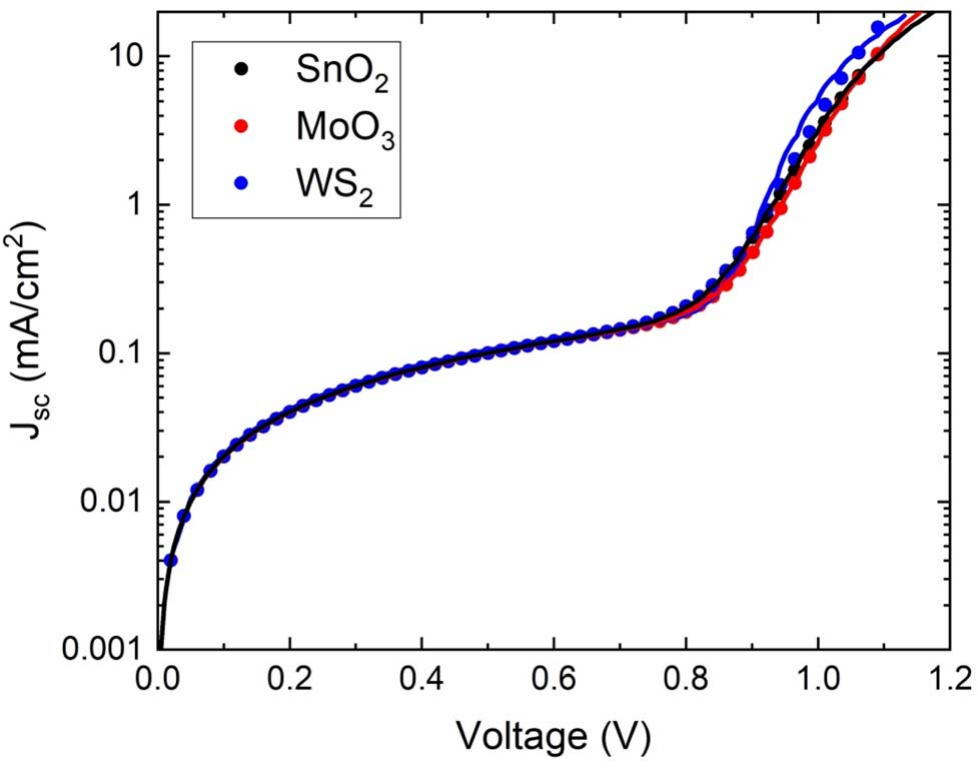
Dark *J*–*V* characteristics of the perovskite solar cells used in model 1.

The analysis of dark *J*–*V* for a photovoltaic device is an indispensable tool to realize the electrical characteristics in favor of the photovoltaic application. In the p–n junction, as a foundational cause of electronic noise, the dark current has been weighed.^56^ As a consequence, the performance of the device was interjected. In the p–n junction, the dark current is indicated as the sum of the components of bulk and surface current.^57^ The tunneling current, diffusion current, and generation–recombination current are part of the bulk component. The leakage shunt current and generation–recombination current are the components of the surface current, which in general appear at the interface of the semiconductor and dielectric devices.^57^ The essence of the electrical properties of the PSC device can be deciphered by fitting dark *J*–*V* characteristics into a single p–n junction model. Fig. 11 shows the dark *J*–*V* curves of the optimized perovskite solar cell. The saturated current (*I*_0_), series resistance (*R*_s_), shunt resistance (*R*_sh_) and ideality factor (*n*) are approximated by fitting the following equation onto the *J*–*V* characteristics of the proposed device.
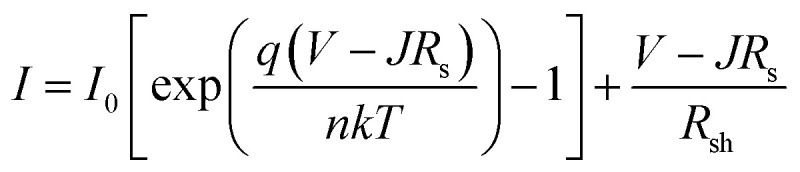
where *k*, *q*, and *T* are Boltzmann's constant, electronic charge, and temperature, respectively. The dark *J*–*V* curve has been plotted by utilizing the simulated data in which the data of current density are applied in logarithmic form (as shown in Figure). The diode characteristics, such as *I*_0_, *R*_s_, *R*_sh_, and *n* of the three different solar cells were extrapolated, as shown in Table 5 by curve fitting using Origin 2018. It can be seen that the lowest saturated current density (*I*_0_) of 1.09 × 10^−14^ mA cm^−2^ was found for the WS2; this lower value signifies that recombination (defect density) in the depletion region is quite insignificant^58^ in this structure.

**Table 5:** Diode characteristics of three different ETL with perovskite structure FTO/ETL/FA_0.85_Cs_0.15_Pb(I_0.85_Br_0.15_)_3_/MoO_3_/Au

ETL	Saturation current density, *I*_0_ (mA cm^−2^)	Ideality factor, *n*	Shunt resistance, *R*_sh_ (Ω cm^2^)	Series resistance, *R*_s_ (Ω cm^2^)
SnO_2_	4.08 × 10^−13^	1.38	4.96	0.00861
MoO_3_	7.31 × 10^−13^	1.36	4.99	0.00881
WS_2_	1.09 × 10^−14^	1.10	4.78	0.00797

Moreover, the lowest series resistance 0.00797 Ω cm^2^ for WS_2_ is attributed to the superior charge transfer capability among the other ETL materials.^59^ The ideality factor (*n*) of the device is a degree of assessment of how much a device is devoid of the ideal diode equation. The assessed ideality factors 1.38, 1.36 and 1.10 have been found for the SnO_2_, MoO_3,_ and WS_2_, which is the implication for the finer PCE and FF of the proposed solar cell. Finally, from the analysis of the dark *J*–*V* curve, the reasons behind the excellent photovoltaic parameters of our proposed structure are unraveled.

## 4. Conclusions

In our current study, compatible ETL and HTL for the MPL titled as FA_0.85_Cs_0.15_Pb (I_0.85_Br_0.15_)_3_ were explored by utilizing multiple ETLs such as SnO_2,_ PCBM, TiO_2_, ZnO, CdS, WO_3_, and WS_2_, and HTLs such as Spiro-OMeTAD, P3HT, CuO, Cu_2_O, CuI, and MoO_3._ Firstly, SnO_2_ as ETL and Spiro-OMeTAD as HTL with the MPL were employed. Utilization of FTO as TCO and Au as a back metal electrode was performed to accomplish the structure as well. By keeping in mind the pragmatic factors, amphoteric defects (2 × 10^15^ cm^−3^), interfacial defects (2 × 10^10^ cm^−2^ and 2 × 10^11^ cm^−2^ at HTL/MPL and MPL/ETL, respectively) and resistance (*R*_s_ = 3 Ω cm^2^ and *R*_sh_ = 5000 Ω cm^2^) were additionally placed in the structure along with IIPPs, which were theoretically and experimentally proven. The simulated results were *V*_OC_= 0.96 V, *J*_SC_ = 20.03 mA cm^−2^, FF = 53.84%, and PCE = 14.12%, which was harmonious with the experimentally substantiated data that also validate our simulation process. While the rest of the parameters and layers were kept invariable, with the variation of ETL and HTL, 42 structures were configured. S4, S6, S16, S18, S22, S24, S28, S30, S34, S36, S40, and S42 were picked out as propitious structures based on performance where S42 was the finest ones among all in which WS_2_ and MoO_3_ were applied as ETL and HTL, respectively. The obtained PPs for the structure S42 were *V*_OC_=1.09 V, *J*_SC_ = 20.71 mA cm^−2^, FF = 74.93% and PCE = 23.10%. With the variation of the thickness of MPL, ETL, and HTL, bulk defects of MPL, and interfacial trap density, this structure was optimized. The optimized thicknesses of ETL, HTL, and MPL were 50 nm, 50 nm, and 450 nm, respectively. In addition, the tolerable bulk defects of MPL, interfacial defects of HTL/MPL, and MPL/ETL were assessed at 1 × 10^14^ cm^−3^, 1 × 10^13^ cm^−2^ and 1 × 10^15^ cm^−2^, respectively. The optimized PPs were *V*_OC_=1.07 V, *J*_SC_ = 21.83 mA cm^−2^, FF = 73.41 and PCE = 23.39%. We performed the dark *J*–*V* analysis to unravel the reasons for the excellent photovoltaic parameters for the optimized structure of PSCs. From the dark *J*–*V* analysis, lower current density and series resistance were observed, which is expectable for good solar cells. The ideality factor was assessed at 1.1, which attests to the better PCE and FF of the PSC. Monitoring of the QE of the structure was carried out by modifying the thickness and bulk defects of the MPL, and on average 88.5% QE was detected under visible light. The scanning of the *C*–*V* and Mott–Schottky plot was fulfilled by the modification of *N*_D_. A declining nature of built-in potential and an increasing behavior in capacitance with the growth of *N*_D_ were observed. In the end, the assessment of the effect of hysteresis on the performance of the PSC was executed. Furthermore, the negative impact of hysteresis was investigated on the performance for model-2, and the decline of *J*_SC_ (76.94%) and PSC (75.4%) was noted, however, no hysteresis effect was noticed for model-1. For annulling this effect, the defect type should be neutral in the MoO_3_/MPL and MPL/WS_2_ interfaces as well as in MPL. With the command over the aforementioned parameters, remarkably efficient, as well as naturalistic PSC FTO/WS_2_/FA_0.85_Cs_0.15_Pb(I_0.85_Br_0.15_)_3_/MoO_3_/Au can be fabricated.

## Data availability

Data will be available from the corresponding author upon request.

## Author contributions

The conceptualization and simulation were carried out by M H Miah and M B Rahman. M H Miah, M B Rahman, and Noor-E-Ashrafi led the manuscript writing effort, with support from M A Islam and M U Khandaker. Review and editing supported by M A Islam. M A Islam supervised the research. The findings were discussed by all contributors, and they all contributed to the final manuscript.

## Conflicts of interest

There are no conflicts of interest.

## Supplementary Material

RA-013-D3RA02170J-s001
