# Supplementary material for: Selection of a compatible electron transport layer and hole transport layer for the mixed perovskite FA0.85Cs0.15Pb (I0.85Br0.15)3, towards achieving novel structure and high-efficiency perovskite solar cells: a detailed numerical study by SCAPS-1D
Source: RSC Adv. 2023 Jun 7;13(25):17130–42. doi: 10.1039/d3ra02170j (PMC10246436; doi:10.1039/d3ra02170j)
Supplement: RA-013-D3RA02170J-s001 [file RA-013-D3RA02170J-s001.pdf]

Electronic Supplementary Material (ESI) for RSC Advances.
This journal is © The Royal Society of Chemistry 2023

Electronic supplementary information (ESI)

Selection of Compatible Electron Transport Layer and Hole Transport Layer for the mixed perovskite $FA_{0.85}Cs_{0.15}Pb(I_{0.85}Br_{0.15})_3$, Towards Achieving Novel Structure and High-efficiency Perovskite Solar Cells: A Detailed Numerical Study by SCAPS-1D

Md. Bulu Rahman[1], Noor–E-Ashrafi[1], Md. Helal Miah[1*], Mayeen Uddin Khandaker[2], Mohammad Aminul Islam[3,*]

[1]Department of Physics, Bangabandhu Sheikh Mujibur Rahman Science and Technology University, 8100 Gopalganj, Bangladesh
[2]Centre for Applied Physics and Radiation Technologies, School of Engineering and Technology, 12 Sunway University, 47500 Bandar Sunway, Selangor, Malaysia
[3]Department of Electrical Engineering, Faculty of Engineering, University of Malaya, Jalan Universiti, 50603 Kuala Lumpur, Malaysia
*Corresponding author: aminul.islam@um.edu.my; helalphydu@bsmrstu.edu.bd

**Table TSI 01:** 42 Structures and their PPs				
Name of the structure	V_{oc} (V)	J_{sc} (mA/cm^2)	FF (%)	η (%)
FTO/ SnO_2/ $FA_{0.85}Cs_{0.15}Pb$ ($I_{0.85}Br_{0.15}$)$_3$/ Spiro- OMeTAD/Au (S1)	0.96	20.09	53.84	14.12
FTO/ SnO_2/ $FA_{0.85}Cs_{0.15}Pb$ ($I_{0.85}Br_{0.15}$)$_3$/ P3HT /Au (S2)	0.94	12.15	24.28	3.76
FTO/ SnO_2/ $FA_{0.85}Cs_{0.15}Pb$ ($I_{0.85}Br_{0.15}$)$_3$/ CuO /Au (S3)	0.85	23.23	58.68	15.73
FTO/ SnO_2/ $FA_{0.85}Cs_{0.15}Pb$ ($I_{0.85}Br_{0.15}$)$_3$/ Cu_2O /Au (S4)	1.09	20.42	64.60	19.64
FTO/ SnO_2/ $FA_{0.85}Cs_{0.15}Pb$ ($I_{0.85}Br_{0.15}$)$_3$/ CuI /Au (S5)	1.01	20.13	56.67	15.67
FTO/ SnO_2/ $FA_{0.85}Cs_{0.15}Pb$ ($I_{0.85}Br_{0.15}$)$_3$/ MoO_3/Au (S6)	1.08	20.19	68.84	20.40
FTO/PCBM/ $FA_{0.85}Cs_{0.15}Pb$ ($I_{0.85}Br_{0.15}$)$_3$/ Spiro- OMeTAD/Au (S7)	0.96	16.61	29.39	6.37
FTO/PCBM/ $FA_{0.85}Cs_{0.15}Pb$ ($I_{0.85}Br_{0.15}$)$_3$/ P3HT /Au (S8)	0.93	8.57	29.36	3.19
FTO/PCBM/ $FA_{0.85}Cs_{0.15}Pb$ ($I_{0.85}Br_{0.15}$)$_3$/ CuO /Au (S9)	0.85	18.25	29.45	6.23
FTO/PCBM/ $FA_{0.85}Cs_{0.15}Pb$ ($I_{0.85}Br_{0.15}$)$_3$/ Cu_2O /Au (S10)	1.09	19.36	30.68	8.83
FTO/PCBM/ $FA_{0.85}Cs_{0.15}Pb$ ($I_{0.85}Br_{0.15}$)$_3$/ CuI /Au (S11)	1.01	17.52	29.16	7.01
FTO/PCBM/ $FA_{0.85}Cs_{0.15}Pb$ ($I_{0.85}Br_{0.15}$)$_3$/ MoO_3 /Au (S12)	1.08	19.86	30.71	8.95
FTO/ TiO_2/ $FA_{0.85}Cs_{0.15}Pb$ ($I_{0.85}Br_{0.15}$)$_3$/ Spiro- OMeTAD /Au (S13)	0.97	20.18	59.87	16.00
FTO/ TiO_2/ $FA_{0.85}Cs_{0.15}Pb$ ($I_{0.85}Br_{0.15}$)$_3$/ P3HT /Au (S14)	0.95	13.16	24.65	4.20
FTO/ TiO_2/ $FA_{0.85}Cs_{0.15}Pb$ ($I_{0.85}Br_{0.15}$)$_3$/ CuO /Au (S15)	0.84	23.61	70.75	19.19
FTO/ TiO_2/ $FA_{0.85}Cs_{0.15}Pb$ ($I_{0.85}Br_{0.15}$)$_3$/ Cu_2O /Au (S16)	1.11	20.47	70.58	21.77
FTO/ TiO_2/ $FA_{0.85}Cs_{0.15}Pb$ ($I_{0.85}Br_{0.15}$)$_3$/ CuI /Au (S17)	1.022	20.20	62.63	17.60
FTO/ TiO_2/ $FA_{0.85}Cs_{0.15}Pb$ ($I_{0.85}Br_{0.15}$)$_3$/ MoO_3 /Au (S18)	1.09	20.22	75.13	22.58
FTO/ ZnO / $FA_{0.85}Cs_{0.15}Pb$ ($I_{0.85}Br_{0.15}$)$_3$/ Spiro- OMeTAD/Au (S19)	0.97	20.18	59.87	16.00
FTO/ ZnO / $FA_{0.85}Cs_{0.15}Pb$ ($I_{0.85}Br_{0.15}$)$_3$/ P3HT /Au (S20)	0.95	13.16	24.65	4.20
FTO/ ZnO / $FA_{0.85}Cs_{0.15}Pb$ ($I_{0.85}Br_{0.15}$)$_3$/ CuO /Au (S21)	0.84	23.61	70.75	19.19
FTO/ ZnO / $FA_{0.85}Cs_{0.15}Pb$ ($I_{0.85}Br_{0.15}$)$_3$/ Cu_2O /Au (S22)	1.11	20.47	70.58	21.77
FTO/ ZnO / $FA_{0.85}Cs_{0.15}Pb$ ($I_{0.85}Br_{0.15}$)$_3$/ CuI /Au (S23)	1.02	20.20	62.63	17.60
FTO/ ZnO / $FA_{0.85}Cs_{0.15}Pb$ ($I_{0.85}Br_{0.15}$)$_3$/ MoO_3/Au (S24)	1.09	20.22	75.13	22.58
FTO/ CdS / $FA_{0.85}Cs_{0.15}Pb$ ($I_{0.85}Br_{0.15}$)$_3$/ Spiro- OMeTAD /Au (S25)	0.97	20.25	59.55	15.95
FTO/ CdS / $FA_{0.85}Cs_{0.15}Pb$ ($I_{0.85}Br_{0.15}$)$_3$/ P3HT /Au (S26)	0.95	13.08	24.59	4.16
FTO/ CdS / $FA_{0.85}Cs_{0.15}Pb$ ($I_{0.85}Br_{0.15}$)$_3$/ CuO /Au (S27)	0.84	23.63	70.69	19.20
FTO/ CdS / $FA_{0.85}Cs_{0.15}Pb$ ($I_{0.85}Br_{0.15}$)$_3$/ Cu_2O /Au (S28)	1.11	20.51	70.31	21.70
FTO/ CdS / $FA_{0.85}Cs_{0.15}Pb$ ($I_{0.85}Br_{0.15}$)$_3$/ CuI /Au (S29)	1.02	20.27	62.32	17.56
FTO/ CdS / $FA_{0.85}Cs_{0.15}Pb$ ($I_{0.85}Br_{0.15}$)$_3$/ MoO_3/Au (S30)	1.09	20.30	75.03	22.60
FTO/ WO_3/ $FA_{0.85}Cs_{0.15}Pb$ ($I_{0.85}Br_{0.15}$)$_3$/ Spiro- OMeTAD/Au (S31)	0.97	20.21	59.89	16.03
FTO/ WO_3/ $FA_{0.85}Cs_{0.15}Pb$ ($I_{0.85}Br_{0.15}$)$_3$/ P3HT /Au (S32)	0.95	13.17	24.66	4.21
FTO/ WO_3/ $FA_{0.85}Cs_{0.15}Pb$ ($I_{0.85}Br_{0.15}$)$_3$/ CuO /Au (S33)	0.84	23.62	70.79	19.21
FTO/ WO_3/ $FA_{0.85}Cs_{0.15}Pb$ ($I_{0.85}Br_{0.15}$)$_3$/ Cu_2O /Au (S34)	1.11	20.48	70.59	21.79
FTO/ WO_3/ $FA_{0.85}Cs_{0.15}Pb$ ($I_{0.85}Br_{0.15}$)$_3$/ CuI /Au (S35)	1.02	20.22	62.64	17.63
FTO/ WO_3/ $FA_{0.85}Cs_{0.15}Pb$ ($I_{0.85}Br_{0.15}$)$_3$/ MoO_3/Au (S36)	1.09	20.25	75.12	22.61
FTO/ WS_2/ $FA_{0.85}Cs_{0.15}Pb$ ($I_{0.85}Br_{0.15}$)$_3$/ Spiro- OMeTAD/Au (S37)	0.97	20.66	59.69	16.36
FTO/ WS_2/ $FA_{0.85}Cs_{0.15}Pb$ ($I_{0.85}Br_{0.15}$)$_3$/ P3HT /Au (S38)	0.95	13.28	24.63	4.24
FTO/ WS_2/ $FA_{0.85}Cs_{0.15}Pb$ ($I_{0.85}Br_{0.15}$)$_3$/ CuO /Au (S39)	0.85	23.78	71.16	19.45
FTO/ WS_2/ $FA_{0.85}Cs_{0.15}Pb$ ($I_{0.85}Br_{0.15}$)$_3$/ Cu_2O /Au (S40)	1.11	20.87	70.41	22.16
FTO/ WS_2/ $FA_{0.85}Cs_{0.15}Pb$ ($I_{0.85}Br_{0.15}$)$_3$/ CuI /Au (S41)	1.02	20.68	62.47	18.00
FTO/ WS_2/ $FA_{0.85}Cs_{0.15}Pb$ ($I_{0.85}Br_{0.15}$)$_3$/ MoO_3/Au (S42)	1.09	20.71	74.93	23.10

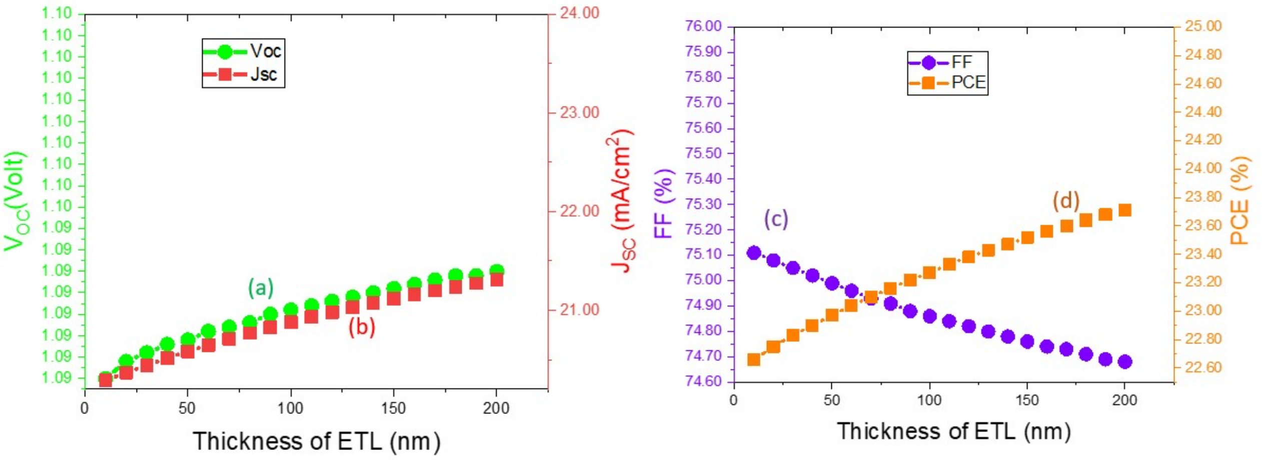

Figure FSI 01 : Variation of PPs owing to changing of ETL thickness

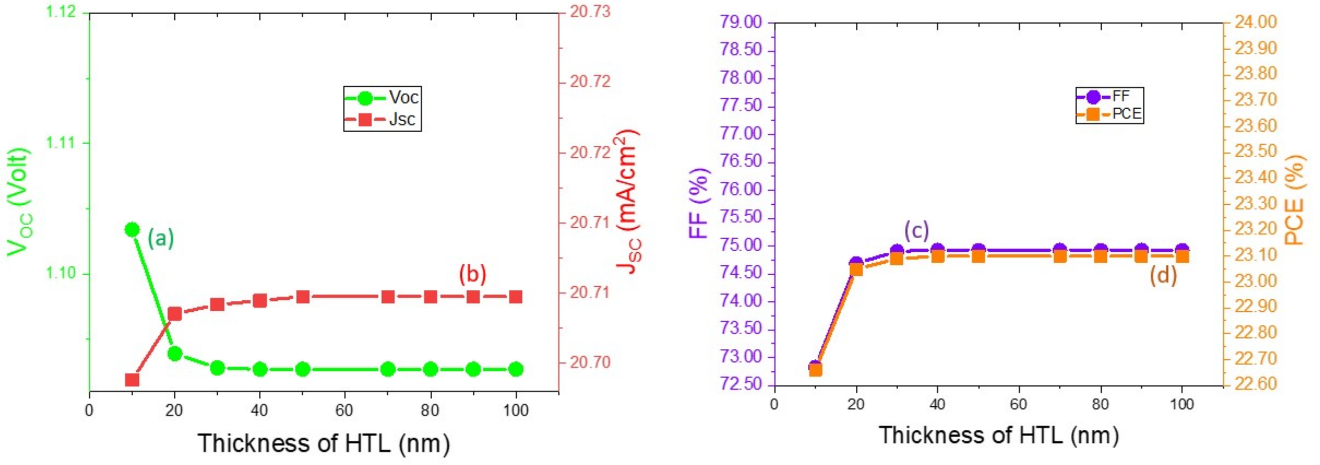

Figure FSI 02 : Alteration of PPs due to changing of HTL thickness
